# Gamma knife capsulotomy for intractable OCD: Neuroimage analysis of lesion size, location, and clinical response

**DOI:** 10.1038/s41398-023-02425-2

**Published:** 2023-04-26

**Authors:** N. C. R. McLaughlin, J. F. Magnotti, G. P. Banks, P. Nanda, M. Q. Hoexter, A. C. Lopes, M. C. Batistuzzo, W. F. Asaad, C. Stewart, D. Paulo, G. Noren, B. D. Greenberg, P. Malloy, S. Salloway, S. Correia, Y. Pathak, J. Sheehan, R. Marsland, A. Gorgulho, A. De Salles, E. C. Miguel, S. A. Rasmussen, S. A. Sheth

**Affiliations:** 1 Butler Hospital, Providence, RI USA; 2 Alpert Medical School of Brown University, Providence, RI USA; 3 Department of Neurosurgery, Perelman School of Medicine, University of Pennsylvania, Philadelphia, PA USA; 4 Columbia University Medical Center, New York, NY USA; 5 Faculdade de Medicina, Universidade de São Paulo, São Paulo, SP Brazil; 6 Department of Methods and Techniques in Psychology, Pontifical Catholic University, São Paulo, SP Brazil; 7 Rhode Island Hospital, Providence, RI USA; 8 Boston University School of Public Health, Boston, MA USA; 9 Providence Veterans Affairs Medical Center, Providence, RI USA; 10 University of Virginia, Charlottesville, VA USA; 11 Department of Neurosurgery, Baylor College of Medicine, Houston, TX USA

**Keywords:** Predictive markers, Psychiatric disorders

## Abstract

Obsessive-compulsive disorder (OCD) affects 2–3% of the population. One-third of patients are poorly responsive to conventional therapies, and for a subgroup, gamma knife capsulotomy (GKC) is an option. We examined lesion characteristics in patients previously treated with GKC through well-established programs in Providence, RI (Butler Hospital/Rhode Island Hospital/Alpert Medical School of Brown University) and São Paulo, Brazil (University of São Paolo). Lesions were traced on T1 images from 26 patients who had received GKC targeting the ventral half of the anterior limb of the internal capsule (ALIC), and the masks were transformed into MNI space. Voxel-wise lesion-symptom mapping was performed to assess the influence of lesion location on Y-BOCS ratings. General linear models were built to compare the relationship between lesion size/location along different axes of the ALIC and above or below-average change in Y-BOCS ratings. Sixty-nine percent of this sample were full responders (≥35% improvement in OCD). Lesion occurrence anywhere within the targeted region was associated with clinical improvement, but modeling results demonstrated that lesions occurring posteriorly (closer to the anterior commissure) and dorsally (closer to the mid-ALIC) were associated with the greatest Y-BOCS reduction. No association was found between Y-BOCS reduction and overall lesion volume. GKC remains an effective treatment for refractory OCD. Our data suggest that continuing to target the bottom half of the ALIC in the coronal plane is likely to provide the dorsal–ventral height required to achieve optimal outcomes, as it will cover the white matter pathways relevant to change. Further analysis of individual variability will be essential for improving targeting and clinical outcomes, and potentially further reducing the lesion size necessary for beneficial outcomes.

## Introduction

Obsessive-compulsive disorder (OCD) affects 2–3 percent of the world population [1] and is associated with major impairment and suffering [1]. Its intrusive, anxiety-provoking obsessions and ritualized compulsions are distressing and can be disabling. It typically has an early age of onset, chronic course, and low rate of remission, factors that in combination contribute to high levels of economic burden, suicidality, and premature death [2, 3]. First-line treatments for OCD include pharmacological and cognitive behavioral therapy, but ~20 percent of patients have refractory illness [4]. These individuals typically have chronic, severe, and disabling symptoms, despite all available conventional and augmenting treatments [4, 5]. For a subgroup of such OCD patients, neurosurgery is a recognized and evidence-based therapeutic option [6].

Neurosurgical options for severe, refractory OCD include stereotactic ablation (anterior cingulotomy, anterior capsulotomy, limbic leucotomy) and deep brain stimulation (DBS) [7, 8]. The therapeutic basis for these procedures derives from interruption or modulation of the aberrant activity known to exist in prefrontal-subcortical circuitry (e.g., anterior cingulate cortex (ACC), orbitofrontal cortex (OFC), and caudate nucleus) in OCD [9]. Neuroimaging studies have found changes in these circuits with pharmacotherapy and psychotherapy [10–13], demonstrating the important role they play in the pathogenesis of OCD. Capsulotomy, for example, consists of lesioning the white matter pathways in the anterior limb of the internal capsule (ALIC) that connect the prefrontal cortex to the thalamus and basal ganglia [9]. Consistent with the mechanisms mentioned above, this procedure produces normalization of metabolism in the ACC, OFC, and caudate that correlates with clinical improvement [14].

Leksell pioneered the use of stereotactic radiosurgery, such as Gamma Knife (GK) protocols, for creating targeted lesions, some advantages of which include the ability to create lesions without a surgical incision and physical brain penetration [15]. Since its inception in the early 1950s, several groups have used radiosurgical capsulotomy to treat OCD. Symptomatic response, defined as a ≥35% decrease in the Yale–Brown Obsessive Compulsive Scale (Y-BOCS) score, is typically observed in 50–60% of patients; [16–19].

As with any stereotactic procedure, the outcome profile of GK capsulotomy (GKC) depends to a great degree on neuroanatomical targeting. GKC targeting has evolved over the decades, largely based on empirical observations. One trend has been the focus on targeting the more ventral portion of the ALIC, rather than its entire height in the coronal plane, as had been done in the original series. This so-called “gamma ventral capsulotomy” (GVC) has retained similar efficacy, but with an improved side effect profile [4, 20]. One unique sham-controlled randomized study has provided further evidence in favor of GVC [16]. Nevertheless, because these procedures are relatively uncommon, even at experienced centers, progress regarding the optimization of lesion targeting remains limited.

To address this limitation, we applied a quantitative approach to analyze post-surgical structural imaging and clinical data to understand the effect of variations in size and location of ALIC targeting on clinical outcome (i.e. change in OCD symptoms). By combining data from two experienced OCD treatment centers that have conducted these procedures for many years, we assembled a cohort large enough for statistical analysis. We examined the size and location of GVC lesions relative to symptomatic outcomes to improve our understanding of the neurocircuitry relevant to a clinical change. In this manner, we sought to maximally benefit from historical experience in order to inform future targeting strategies and improve outcomes. Based on prior research, we hypothesized that the bottom half of the internal capsule in the coronal plane would be the most efficacious target to produce OCD symptom reduction.

## Materials and methods

### Participants

Both participating institutions obtained IRB approval to share and collectively analyze the data, and informed consent was collected from participants. We retrospectively analyzed data from 26 patients (9 female, 17 male) who had undergone bilateral GVC for severe and treatment-refractory OCD. Selection criteria to receive GVC included a history of severe, unremitting OCD despite treatment with evidence-based exposure and response prevention methods (>20 h of 1:1 intervention or inability to engage in therapy due to severity of illness) and multiple pharmacological trials (serotonin reuptake inhibitor (SRI) and clomipramine trials, clonazepam, and augmentation with a neuroleptic; see refs. [16, 17] for detailed information).

Requirements for inclusion in this retrospective targeting-outcome study were the availability of follow-up clinical and imaging data. The procedures were performed between 2003 and 2014 at the University of São Paulo (USP; *n* = 14) or Butler Hospital/Rhode Island Hospital/Alpert Medical School of Brown University (BH/RIH; *n* = 12). The 14 participants from USP included 11 of the 12 subjects from their previously reported randomized clinical trial (RCT) [21] and 3 of the 5 participants from their earlier pilot trial [20]. The one RCT and two pilot trial participants not included in this current analysis were excluded because follow-up imaging was not available. The 12 participants from BH/RIH were patients from the clinical sample [17] who were able to come back for long-term follow-up.

### Radiosurgical procedure

Radiosurgical targeting was performed consistently between the two institutions, as originally developed at BH/RIH and adopted at USP [16, 17]. Targeting at USP was defined together by members of USP and BH/RIH. Ongoing collaboration and frequent communication between the institutions over this period ensured consistency. Procedures were performed on the Gamma Knife model U and model C at Rhode Island Hospital and model B at Santa Paula Hospital (affiliated with USP). The GVC radiosurgical plan consisted of two 4-mm isocenters placed in a vertically stacked configuration bilaterally (“double shot”). In the axial plane, the isocenters were located within the ALIC, 8–10 mm anterior to the posterior border of the anterior commissure. In the coronal plane, the ventral isocenter of the double-shot pair was placed in the ventral portion of the capsule, bordering the superior aspect of the ventral striatum. In the same plane, the dorsal border of the dorsal shot of the pair was typically near the mid-portion of the capsule, directly superjacent to the ventral shot. Thus the intended lesion, within the 50% isodose line, occupied the ventral one-third to one-half of the capsule. The prescription dose was 90 Gy to the 50% isodose point, thus 180 Gy to the maximum isodose point.

### Imaging and clinical data

At follow-up appointments, patients underwent high-resolution T1 MRI sequences on a 1.5T (USP) or 3T (BH/RIH) scanner. The severity of patients’ OCD symptoms was evaluated using the Yale–Brown Obsessive Compulsive Scale (Y-BOCS) and was tracked as a percent reduction from pre-operative levels [22].

**Fig. 1:**
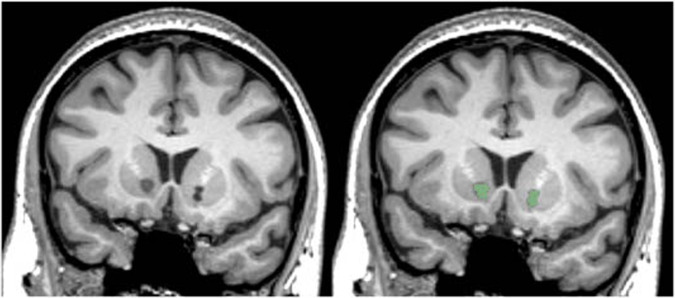
Bilateral capsulotomy lesions. *Left*. A coronal slice of bilateral capsulotomy lesions in native space. *Right*. The hand-traced mask (green) for this slice.

**Fig. 2:**
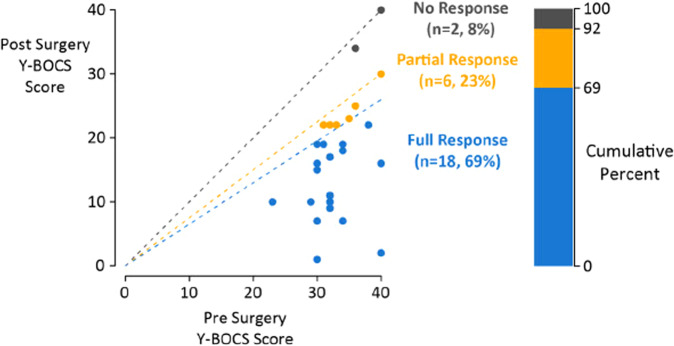
Change in Y-BOCS scores following surgery. Each point shows a single patient’s pre-treatment (*x*-axis) and post-treatment (*y*-axis) Y-BOCS scores. Dashed lines show cutoffs for clinical categories: full response (35% reduction; blue), partial response (at least 25% reduction; yellow), and no response (<25% reduction; black). The inset stacked bars provide cumulative percentages of each category.

Lesions were delineated by two raters (GPB, PN), blinded to patient information, who manually traced lesion masks on T1 images using 3D Slicer software [23]. All scans were windowed to the same level, and lesions were traced based on the clear presence of ablated tissue, with local intensity comparable to cerebrospinal fluid (see Fig. 1). Lesion masks were inspected, edited, and approved by an experienced neuropsychologist (NCM), also blinded to patient response status. Inter-rater reliability was evaluated by calculating the Sørensen–Dice index of similarity between the two raters’ sets of lesion masks and by performing a Pearson correlation between total lesion volume. Differences in lesion size between the raters were quantified with the median of the lesion volume difference percentage; for each volume, the difference in size between the raters was scaled by the average of the two sizes [(*v*1−*v*2) / mean(*v*1,*v*2)]. Only voxels judged by both raters as lesioned were included in the final masks. The masks were linearly transformed (using AFNI 3dAllineate) from patients’ scans to the standard MNI152 2009 1 mm template using the transform matrix derived from mapping the subject’s T1 volume to MNI space (using AFNI @auto_tlrc) (Fig. 2).

### Lesion-outcome statistical analysis

For the analysis regarding the association between lesions and clinical outcome, Y-BOCS data from the time point closest to the imaging date were selected for each participant. Data analysis was done in R using the ANTsR, LESYMAP, and lme4 packages. MRI data were normalized and visualized using AFNI.

We first built a linear model estimating post-treatment Y-BOCS scores from pretreatment scores across patients to determine the average change following surgery. This model allowed us to estimate the average Y-BOCS change following surgery. The residual (prediction error) from this model provides an index of how much better (or worse) a given individual performed relative to the average: negative residuals indicate lower than expected post-treatment Y-BOCS scores (better than expected outcome); positive residuals indicate higher than expected post-treatment scores (worse than expected outcome). For all visualizations of these residuals, negative values are plotted “up” so that better-than-average outcomes are higher on the *y*-axis than worse-than-average outcomes.

Next, we built three models to explore the association between lesion location and the residualized Y-BOCS scores. Using the residual Y-BOCS is statistically equivalent to predicting post-treatment Y-BOCS score while adjusting for pre-surgical score. Using the Y-BOCS residual is also more statistically powerful than the binary response/no-response measure, helping to compensate for the small sample size and lack of a control group. Given the heterogeneity of the lesions, we included in the model all voxels from the targeted lesion mask that were damaged in at least 10% of patients (*n* ≥ 3; 1908 total voxels selected). Given the small sample size (*n* = 26), we did not include covariates.

In the first model, we built univariate linear models for each voxel relating lesion status (0 vs. 1) to the Y-BOCS residual. The *t*-score associated with the lesion status predictor was overlaid on the MNI template for visualization. To quantify the spatial patterns revealed by this model, we used univariate regression models to predict the residual Y-BOCS change score as a function of each voxel’s location in 3D space (medial to lateral, anterior to posterior, superior to inferior), separately for each hemisphere.

The strong univariate associations between location and Y-BOCS residual allowed us to develop a multiple regression model that used each voxel’s (median-centered) *x*, *y*, and *z* locations to predict the residual Y-BOCS score (separately for each hemisphere). To determine the best variables and interactions to include in this model, we used the stepwise regression procedure with the Bayes Information Criterion (BIC) method (which allows the model to include interactions after initial specifications) for inclusion/exclusion of terms (the *step* function in R, with *k* = log(*n*) to implement BIC).

To assess the robustness of the mass-univariate model, we fitted a third model that was multivariate across voxels. We used the multivariate lesion-symptom mapping method called sparse canonical correlation analysis for neuroimaging (SCCAN) [24] to learn a sparse mapping from the patient’s lesions to the patient’s residual Y-BOCS scores. SCCAN produces bi-directional voxel weights, allowing the determination of which voxels are associated with better-than-average vs. worse-than-average Y-BOCS scores.

## Results

### Clinical response

Average age at the time of surgery was 32.7 years (SD = 10.3; range 18–55 years). Mean Y-BOCS at baseline was 33.2 (SD = 4.1), representing extreme levels of illness. Of the study’s 26 subjects, 18 (69.2%) were full responders (defined as ≥35% Y-BOCS improvement), 6 (23.1%) were partial responders (defined as 25–34% Y-BOCS improvement), and 2 (7.7%) were non-responders (<25% Y-BOCS improvement) at the follow-up appointment closest in time to the scan date. The interval between the procedure and follow-up MRI scan/Y-BOCS rating was a mean of 62.4 months (SD = 62.7; range = 7–192 months). The mean Y-BOCS reduction at follow-up relative to pre-operative baseline was 49.2% (SD = 24.1%), with a range of 0–96.7%. Confirming this strong pattern of reductions, a linear mixed-effects model with time (pre vs. post) as a fixed effect and patient as random intercept yielded evidence for a significant group-level decrease [mean change = −16.1, *t*(25) = −9.9, *p* = 10^−10^].

### Lesion analysis

The volumes of the raters’ lesion masks were significantly correlated (*r* = 0.85; *p* < 0.001). The median percent difference in volume between the raters’ lesion masks was 26%. The Sørensen–Dice index between the two sets was 0.71, which is considered a “high” degree of spatial similarity [25, 26].

**Fig. 3:**
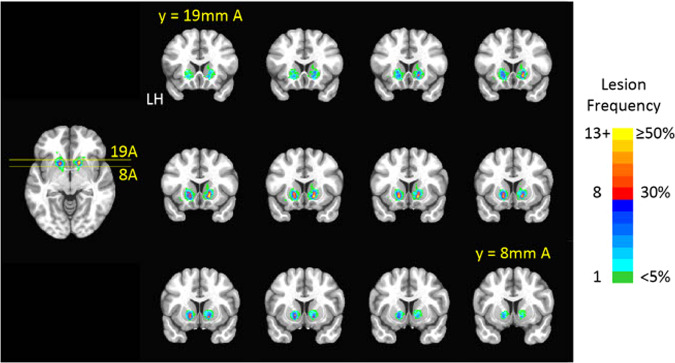
Lesion locations. Heat maps represent the location of lesions linearly transformed onto the standard MNI152 1 mm brain template. Colors indicate lesion frequency for each voxel, in terms of the number (left side of the color bar) or percent (right side of the color bar) of patients. LH: Left hemisphere. Slice labels indicate *y* (anterior–posterior) position in mm anterior (A) to the anterior commissure.

Lesions primarily developed in the ventral portion of the anterior limb of the internal capsule, as expected based on radiosurgical targeting. We also observed some encroachment of the lesioned volumes into adjacent gray matter structures, including the caudate, putamen, and ventral striatum (Fig. 3). It is notable that despite consistent radiosurgical planning, we observed significant variability in the volume of the eventual lesion, likely due to inter-individual variability in radiobiological sensitivity of the brain tissue. The mean lesion volume before transformation to standard space was 440 mm^3^ (SD = 502 mm^3^), with a range of 0–2086 mm^3^.

Although there was generally substantial improvement in Y-BOCS scores across patients, total lesion volume was not a significant predictor of Y-BOCS change scores (effect estimate = −0.002, *p* = 0.62), indicating that simply creating a bigger lesion within the targeted region (ventral portion of the ALIC) did not result in improved outcomes.

**Fig. 4:**
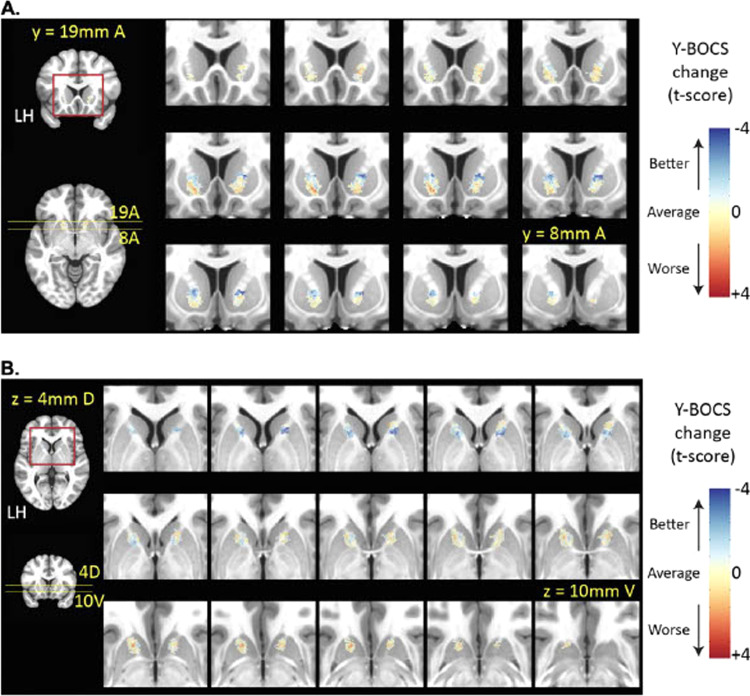
Univariate lesion-symptom maps. **A** Coronal slices (magnified view of the region in the red box) depict univariate lesion-symptom maps, with warmer colors depicting worse than the average change in Y-BOCS *t*-scores, and cooler colors depicting greater than average change. The axial slice shows the anterior-to-posterior extent of the coronal slices. Lesions including the dorsal region of the targeted area (near the middle third of the capsule) were associated with decreased OCD symptomatology as compared to lesions including the most ventral region of the capsule. LH: Left hemisphere. **B** Univariate lesion-symptom maps overlaid on axial slices on a 1 mm MNI template. Slices shown cover from 4 mm superior of AC/PC to 10 mm inferior (magnified view of the region in the red box). Coronal slice shows the superior to the inferior extent of the axial montage. For each voxel, a *t*-test was conducted comparing change in Y-BOCS score between those with and without a lesion at that location. Blue indicates better than average change (lower Y-BOCS, negative *t*-scores); red indicates worse than average change.

To identify lesioned areas associated with better outcomes, we first created univariate lesion-symptom maps for relative improvement in Y-BOCS scores. This voxel-wise approach identified notable spatial patterns in both dorsal–ventral and anterior–posterior directions. In the coronal plane (Fig. 4A), lesions including the dorsal portion of the targeted area (approximately the middle third of the capsule) were related to better than mean improvement on the Y-BOCS; whereas, lesions including the most ventral portion of the capsule were related to lower than mean improvement. In the axial plane (Fig. 4B), decreased OCD symptomatology was associated with lesion development posteriorly in the targeted region, closer to the AC. Lower than the average outcome was associated with more anterior lesions.

**Fig. 5:**
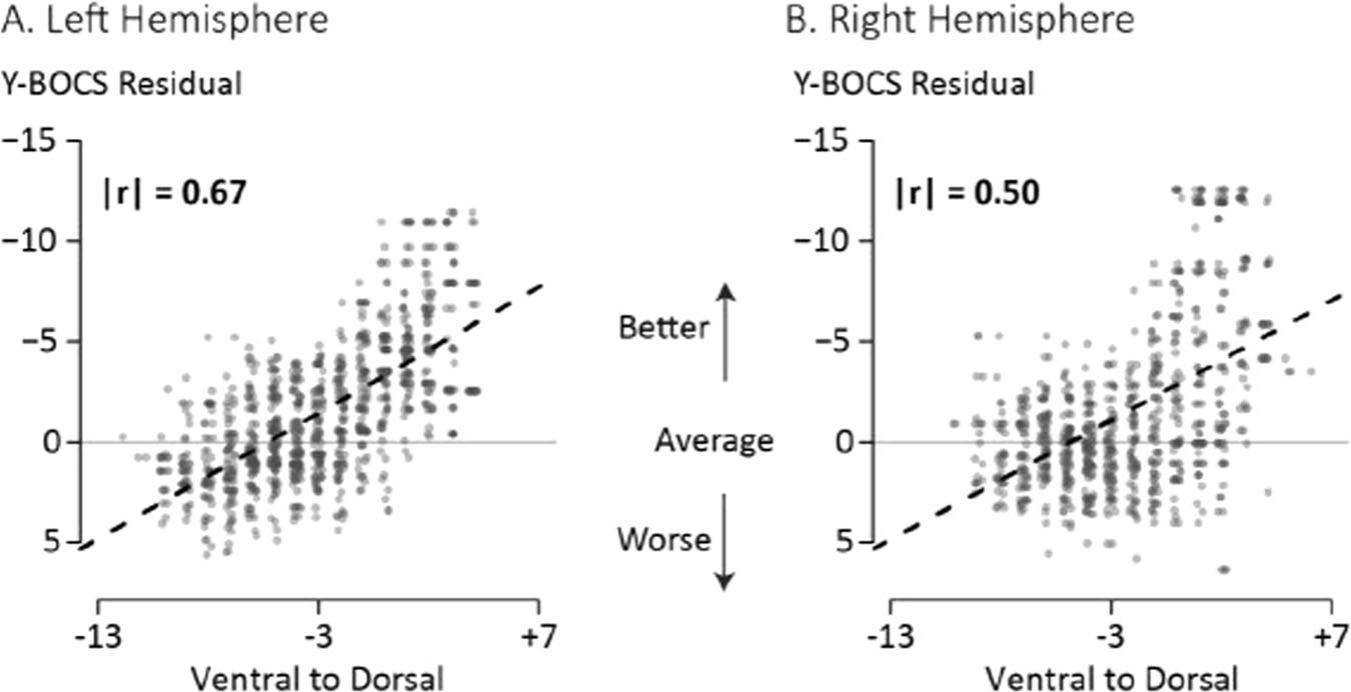
Association between clinical outcome and lesion location along the ventral to dorsal (V–D) axis of the ALIC. **A** Each point represents a lesioned voxel in the left hemisphere, arranged by location along the ventral-to-dorsal (*z*-) axis (value is mm from the AC-PC plane). Dashed line is the linear fit between the slice location and the Y-BOCS residual change score (negative values indicate better than average reduction and are plotted “up”; positive values indicate worse than average reduction). The horizontal line at 0 indicates average Y-BOCS reduction following surgery. Only voxels lesioned in at least three patients are included. Absolute value of the correlation shows the linear relationship between the *z*-axis location and the Y-BOCS residual. **B** The same data for the right hemisphere lesioned voxels.

We quantified the observed dorsal vs. ventral pattern by comparing the average Y-BOCS residual change scores for lesioned voxels with the voxels’ location along the *z*-axis (Supplemental Figures). There were significant linear relationships for both the left (*r* = −0.67, *p* < 0.001) and right (*r* = −0.50, *p* < 0.001) hemispheres: more dorsal locations were associated with more Y-BOCS improvement. As compared to the dorsal/ventral relationships, the magnitude of the correlations along the left-to-right axis, though statistically significant, were smaller (LH: *r* = 0.07; RH: *r* = 0.20; both *p*s < 0.001), as were correlations along the anterior-to-posterior (LH: *r* = 0.17; RH: *r* = −0.22; both *p*s < 0.001) axis. Along all axes, the direction of the correlation was the same across hemispheres, with some variation in the correlation magnitude (Fig. 5).

We used stepwise regression to build the best-fitting multiple linear regression model of these spatial differences (all variables were median-centered prior to fitting) in Y-BOCS residual change scores, separately for each hemisphere. Centered variables were used to aid in interpretation. Table 1 shows the final results for each hemisphere. For the left hemisphere (overall *R*^2^ = 0.49), the final fitted model was straightforward, with the strongest effect along the *z*-axis [dorsal/ventral] (*b* = −0.66, *p* < 0.001), followed by the *x*-axis [right/left] (*b* = −0.27, *p* < 0.001), along with a minor contribution from their interaction (*b* = −0.03, *p* < 0.001). For the right hemisphere (overall *R*^2^ = 0.58), the best-fitting model was a bit more complex, containing significant effects of both the *z*-axis [dorsal/ventral] (*b* = −0.51, *p* < 0.001) and *y*-axis (*b* = −0.25, *p* < 0.001), but not the *x*-axis [right/left] (*b* = −0.04, *p* = 0.372; included in the final model because of its presence in an interaction term). There were also interactions between the *z*- [dorsal/ventral] and *y*-axes [anterior/posterior] (*b* = −0.23, *p* < 0.001) and the *x*- [right/left] and *y*-axes [anterior/posterior] (*b* = −0.17, *p* < 0.001). Taken together, the multiple regression models confirm that better outcomes were associated with lesions in the more dorsal aspect of the targeted region (approximately the middle third of the capsule in the coronal view), with smaller benefits of moving laterally (for the left hemisphere) or posteriorly (for the right hemisphere).

**Table 1:** Best fitting coefficients for multiple linear regression models (fit separately for left and right hemispheres) relating Y-BOCS residual to spatial location, with significant results included in the table.

Hemisphere	Coefficient	Estimate	*t*-value	*p*-value
Left	Intercept	−1.41	−18.5	10^−16^
	*Z*_C_	−0.66	−31.3	10^−^^16^
	*X*_C_	−0.27	−8.9	10^−^^16^
	*Z*_C_ × *X*_C_	−0.03	−3.4	10^−^^3^
Right	Intercept	−0.63	−6.1	10^−9^
	*Z*_C_	−0.51	−16.4	10^−16^
	*Y*_C_	−0.25	−7.5	10^−13^
	*X*_C_	−0.04	−0.9	0.37
	*Z*_C_ × *Y*_C_	−0.23	−21.2	10^−16^
	*X*_C_ × *Y*_C_	0.17	−12.8	10^−16^

Subscript “C” indicates the variable was median-centered prior to fitting. Intercept corresponds to the central lesion location (*X*_C_ = *Y*_C_ = *Z*_C_ = 0). Within each hemisphere, variables are sorted according to the magnitude of their estimated coefficient (Estimate column). Degrees of freedom were 841 (left hemisphere) and 1057 (right hemisphere) for *t*-tests on coefficients.

## Discussion

We pooled data from GKC for treatment-refractory OCD procedures across two leading OCD treatment research centers to understand the relationship between lesion location and symptomatic outcome. Our results demonstrate that lesions including the middle third of the dorsal–ventral extent of the capsule (as viewed in the coronal plane) were associated with the greatest improvement.

### Clinical outcomes

The observed responder rate in our analysis (69%) is consistent with other contemporary GKC studies using a similar targeting and dose strategy [4, 17]. All patients for whom follow-up imaging was available were included, but the subjects in our study from USP were a subset of those included in their group’s previous pilot trial [20] and double-blind, sham-controlled randomized clinical trial [21]. In addition, the BH/RIH patients were a subset of those from a previously reported cohort [17]. Responder rates from those reports were 60% (3/5), 58% (7/12), and 55% (22/40), respectively; thus, while the responder rate in this study was slightly higher than the parent studies, they were comparable.

Numbers of adverse events stemming from capsulotomy procedures have decreased as radiosurgical targeting methods have evolved. Reported adverse events include headache, hypomania, weight gain, and a few others [17]. The most concerning, however, is radiation-induced frontal lobe edema causing frontal cognitive-behavioral dysfunction including apathy, fatigue, disinhibition, memory difficulties, and delirium [17]. An early series from the Swedish group using higher radiation doses (180–200 Gy) and larger lesion volumes (3 or 4 isocenters, each 4 mm, designed to cover the majority of the ALIC) reported symptomatic frontal lobe edema in 5/9 (56%) patients, with long-lasting effects in 3 (33%) [27]. A subsequent series with additional patients from the same group reported the same adverse effects in 4/8 (50%) patients [18]. Further analysis in the latter study demonstrated that higher radiation doses (e.g., ≥3 isocenters bilaterally, >180 Gy maximum dose) or repeat procedures were most likely to produce these adverse events.

Treatment with lower radiation doses and smaller lesions has produced fewer adverse events. The USP pilot trial (2 isocenters, 180 Gy max dose) reported temporary headaches in one patient, with no long-term adverse events [20], and their larger RCT reported frontal edema in one patient that also resolved with no long-term sequelae [21]. Across both sites, there have been four patients who have developed cysts, one of whom required additional neurosurgical treatment to drain and later remove the cyst [16, 17]. Neuropsychological testing of patients in both of these samples demonstrated the stability of all motor and cognitive functions tested and actually showed improvement in some cognitive domains [28–32]. The recent series from Sheehan (1 isocenter, 140–160 Gy) (2013) and Kondziolka (2 isocenters, 140–150 Gy) (2011) [33, 34] also observed no adverse events.

The available evidence, therefore, suggests that lowering radiation dose (whether by decreasing lesion size or maximum dose or both) reduces the adverse event burden. However, there also seems to be a competing trend indicating reduced effectiveness with decreased radiation dose. In the previously reported BH/RIH cohort, though only followed for less than a year on average, a single 4 mm shot resulted in a clinical response in only 1/15 (7%) patients [17]. A multi-center cohort not overlapping with the USP and BH/RIH cohorts also showed that treatments using 2 shots (vs. 1 shot) were associated with significantly improved outcomes [35]. Our group (BH/RIH and USP) has also seen a low responder rate of 0/11 in a recent series of patients treated with a single shot placed in the ventral-most aspect of the ALIC [36, 37], likely due to the ventral placement of the lesion. Even when radiosurgical planning is consistent, as in the data presented here, differences in radiobiology and possibly vascular microanatomy produce variability in lesion size.

### Target optimization

Our results provide an opportunity to evaluate optimal targeting for stereotactic interventions for OCD, whether using ablative procedures or deep brain stimulation (DBS). Just as ablative procedures have focused on the ventral portion of the ALIC, based on empirical evidence and neuroanatomy [4, 17], the DBS field has largely done the same [9]. Better understanding of the optimal target will enable increasingly precise treatments, with larger therapeutic windows and lower adverse event profiles.

The size of the cohort included in this study is large relative to many other psychiatric neurosurgical lesion studies, but it is still limited from a statistical point of view. Because most patients improved symptomatically, and because all were targeted similarly (in the ventral third to half of the ALIC), lesion damage within the targeted region was statistically associated with improvement. But even with this limitation, the natural variation of the spatial development of the lesion produced sufficient variability to observe a dorsal/ventral effect; lesions extending into the dorsal portion of the targeted region, near the middle of the ALIC in coronal view, had a stronger association with improvement.

A tempting test of this association, especially in light of the above discussion on more focused treatment plans, would be to confine the lesion to the mid-region of the ALIC alone, without the more ventral component. But prior experience with this strategy suggested that it was insufficient, as only 1/15 (7%) of patients with a single shot targeted in the mid-ALIC were responders [17]. This was likely due to the size and location of the target in relation to the pathways coursing through that region. Though it is possible there may have been an increase in response with extended follow-up, it is unlikely that a significant number of patients would have moved to responder status. In that sample, adding a ventral lesion to the pre-existing dorsal lesion increased clinical response (including full and partial responders) to 54% of the sample. As mentioned above, targeting the most ventral region alone, with a single 4 mm isocenter, is also likely insufficient. Combining lessons from previous studies with our current results, until we are able to effectively individualize targeting, our interpretation is that an effective lesion likely needs to cover the ventral half of the ALIC.

Beyond the dorsal–ventral consideration, our results also speak to anterior–posterior optimization. At least in the right hemisphere, our model suggested benefit with lesions developing in the posterior aspect of the targeted region, within 8 or fewer mm of the anterior commissure (AC). DBS studies for OCD have empirically observed a similar benefit when targeting close to the AC [9] with some recent series aiming at or even just posterior to the AC in the region of the bed nucleus of the stria terminalis [38, 39]. Lesions will be more difficult to place with very close proximity to the AC-given structures such as the fornices that should not be damaged, but our data show the importance of the concept of proximity to the AC for lesion procedures. This proximity may allow the engagement of critical white matter pathways in a region where they are more compact, before they fan out further anteriorly, as discussed in the next section.

These lessons regarding targeting can be extended beyond radiosurgical methods. Other capsulotomy lesion modalities such as MRI-guided focused ultrasound [40, 41] or laser interstitial thermal therapy [42] will have their own dosing considerations but can benefit from this approach to targeting.

### Network considerations

There is increasing awareness that targeted therapies such as neurosurgical procedures should be thought of as targeting a symptomatic network rather than targeting isolated dysfunctional regions in the brain [43]. From this perspective, our surgical targets are white matter targets, situated at critical hubs or crossroads of pathways projecting to a wider symptomatic network. A well-placed intervention, though narrowly targeted, can thus affect several key regions across the brain. Recent neuroanatomical work has demonstrated the relationship between a number of brain targets used for stereotactic procedures treating OCD, including the ventral ALIC, ventral striatum, subthalamic region, and midbrain tegmentum [44]. Though all four targets engage some unique pathways, one major tract common to all four is the hyperdirect pathway from the orbital and/or dorso-medial prefrontal cortex to the subthalamic nucleus (STN) region. Engagement of analogous hyperdirect connections between the motor cortex and STN is emerging as critical for the response observed in DBS for movement disorders [45].

The region targeted in our study, especially its dorsal aspect (which was most significantly associated with response), is likely proximal to a tract identified as central to symptomatic response in a recent multicenter European study of DBS for OCD [46]. Those authors suggest that this tract contains hyperdirect fibers from the dorsal anterior cingulate cortex (dACC) to STN. Since the dACC is itself an effective lesion target for OCD (i.e., the cingulotomy procedure) [19], the dACC hyperdirect hypothesis provides a parsimonious way to connect interventions in dACC with those in the ventral ALIC.

The ventral aspect of the region targeted in our study contains tracts connecting to ventromedial prefrontal and orbitofrontal cortex, possibly representing analogous hyperdirect pathway fibers from these prefrontal regions. Modulation of this system may be more directly responsible for the mood improvement commonly seen in these patients, as evidenced in prior trials [17]. Tractography studies in DBS for depression have demonstrated the importance of tracts connecting this ventral/medial/orbital cortical region with the ventral midbrain [47, 48]. Indeed, direct electrical stimulation of OFC can produce mood elevation [49]. These converging lines of evidence suggest that tracts in this most ventral region of the capsule may be most responsible for the mood-elevating effects of lesions in this region.

Future work in this field will extend these network concepts beyond the understanding of pathways common to response across patients to an understanding of circuit dysfunction specific to an individual patient. As we better understand the networks that are dysfunctional in OCD [50] and the connections between them [44, 46, 51], this “precision medicine” approach will help further refine targeting for our neurosurgical procedures. This individual specificity will be critical; though the general topology of the organization of the tracts in the ALIC is consistent, prior studies have shown high inter-individual variability regarding the exact location of those tracts within the capsule [51–53].

Prospective studies should aim to assess participants on a longitudinal basis to determine the evolution of network changes over time, in response to stereotactic lesions or DBS. This knowledge could be applied to currently used techniques in order to identify and target specific fiber bundles to improve outcomes. In addition, it may also guide the development of less invasive procedures targeting similar circuitry. For example, current OCD research is using transcranial magnetic stimulation, pulsed ultrasound, and low-dose radiotherapy to produce localized changes in discrete targets without creating lesions. In addition, improved circuit-based interventions, either invasive or non-invasive, might better augment subsequent behavioral therapies that proved ineffective before surgery [43]. These pathways of change are unlikely to be unique to OCD but relevant to other anxiety disorders and mood disorders that share comparable abnormal neurocircuitry.

## Supplementary information

Supplemental Figure S1

Supplemental Figure S2
