# Supplementary material for: Gamma knife capsulotomy for intractable OCD: Neuroimage analysis of lesion size, location, and clinical response
Source: Transl Psychiatry. 2023 Apr 26;13:134. doi: 10.1038/s41398-023-02425-2 (PMC10130137; doi:10.1038/s41398-023-02425-2)
Supplement: Supplementary file 1 — Supplemental Figure S1 [file 41398_2023_2425_MOESM1_ESM.docx]

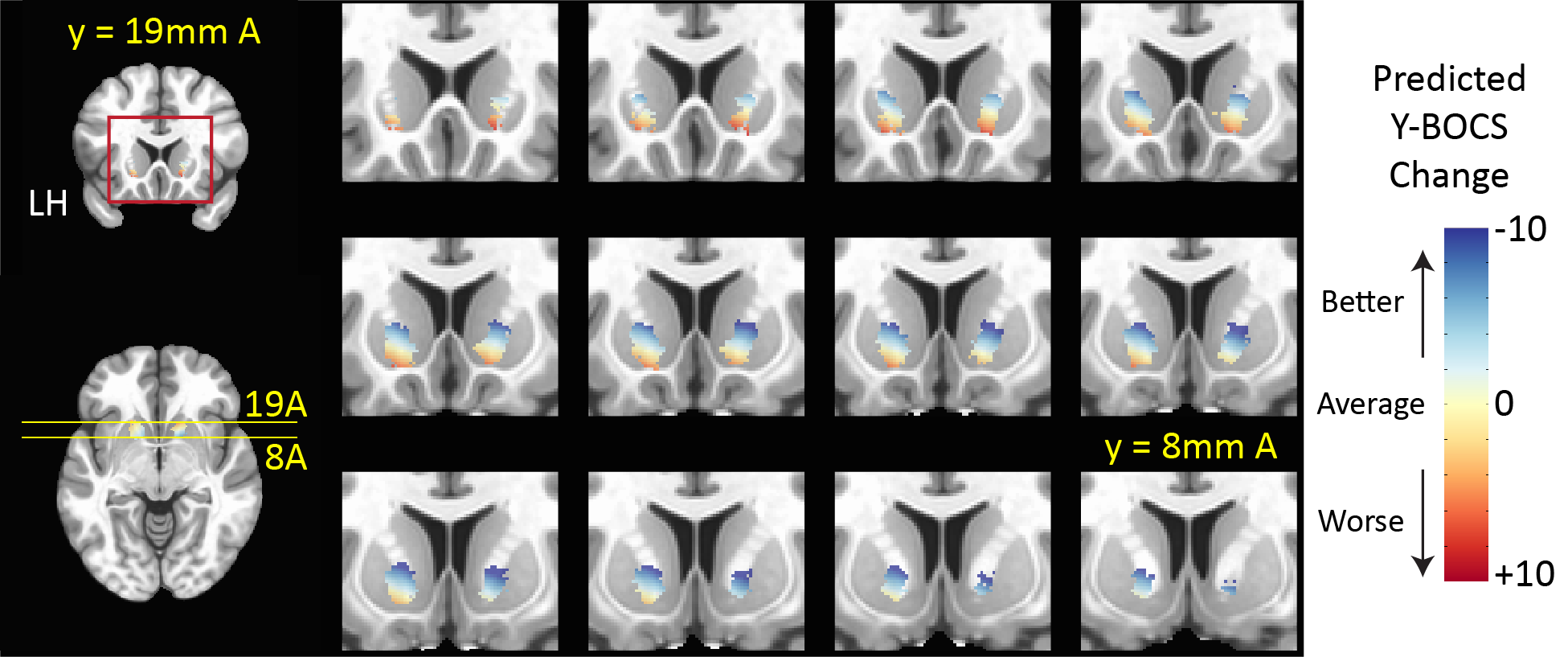


**Supplemental Figure S1.** Coronal slices showing predicted change in Y-BOCS (relative to average change). The color indicates the predicted relative change (blue, lower Y-BOCS) from a regression model that included only the anatomical locations of the damage.
