# Supplementary material for: Gamma knife capsulotomy for intractable OCD: Neuroimage analysis of lesion size, location, and clinical response
Source: Transl Psychiatry. 2023 Apr 26;13:134. doi: 10.1038/s41398-023-02425-2 (PMC10130137; doi:10.1038/s41398-023-02425-2)
Supplement: Supplementary file 2 — Supplemental Figure S2 [file 41398_2023_2425_MOESM2_ESM.docx]

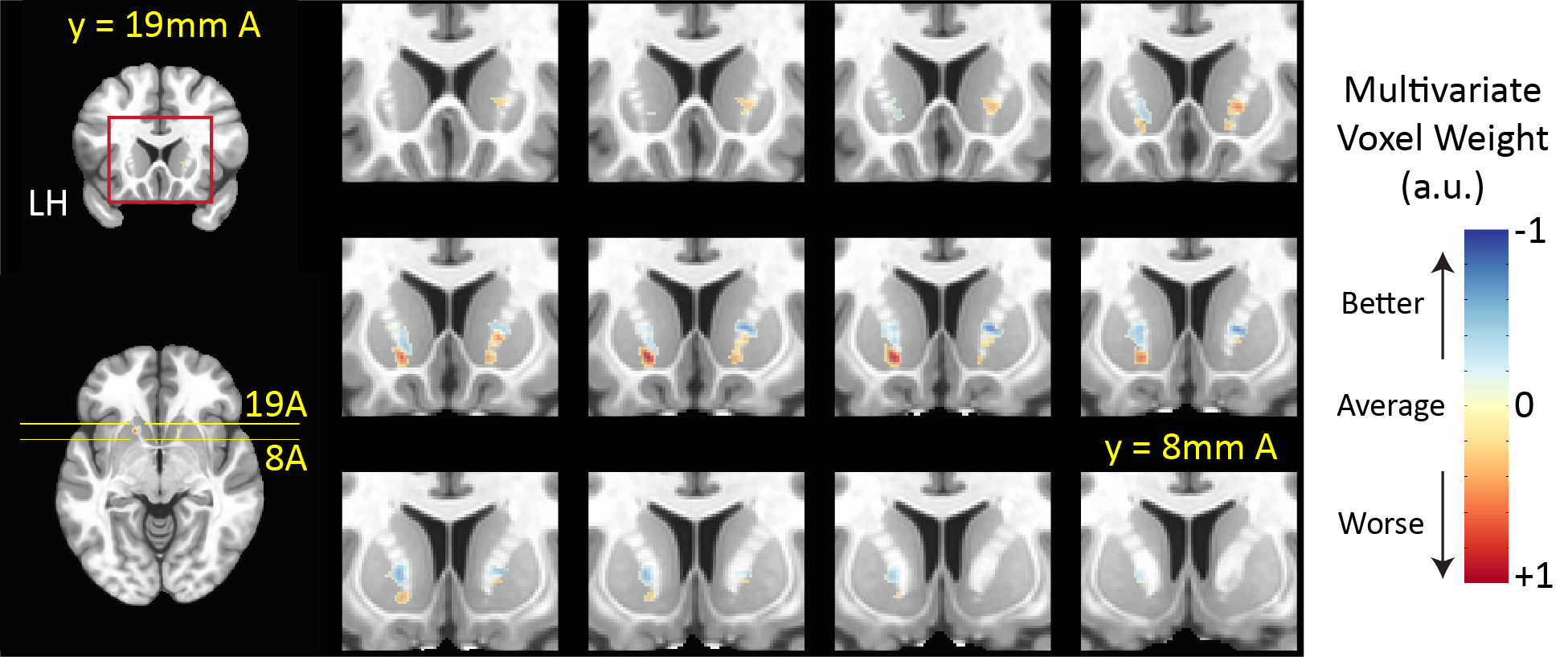


________________________________________________________________________

**Supplemental Figure S2. Multivariate lesion symptom map.** Result of using the SCCAN method to learn the lesion pattern best associated with residual Y-BOCS scores. Voxel color indicates the weight given to each voxel; negative (blue) weights indicate better than average reduction in Y-BOCS scores; positive (red) weights indicate regions associated with worse than average Y-BOCS change scores.
